# Of flies and men: insights on organismal metabolism from fruit flies

**DOI:** 10.1186/1741-7007-11-38

**Published:** 2013-04-15

**Authors:** Akhila Rajan, Norbert Perrimon

**Affiliations:** 1 Department of Genetics, Harvard Medical School, 77 Avenue Louis Pasteur, Boston, MA 02115, USA; 2 Howard Hughes Medical Institute, 77 Avenue Louis Pasteur, Boston, MA 02115, USA

## Abstract

The fruit fly *Drosophila *has contributed significantly to our general understanding of the basic principles of signaling, cell and developmental biology, and neurobiology. However, answers to questions pertaining to energy metabolism have been so far mostly addressed in more complex model organisms such as mice. We review in this article recent studies that show how the genetic tractability and simplicity of *Drosophila *are being used to identify novel regulatory mechanisms at the organismal level, and to query the co-ordination between energy metabolism and other processes such as neurodegeneration, circadian rhythms, immunity, and tumor biology.

## 

Proteins, fats and sugars make up a large proportion of the food we consume. These macromolecules are broken down by evolutionarily conserved biochemical pathways - such as glycolysis, the citric acid cycle and oxidative phosphorylation - to generate the primary energy currency of cells, ATP, and other requisite biomolecules in our body. When nutritional supply exceeds the energy needs, the excess is stored for use at a later time point, such as during starvation, stress or infections. There is a constant flux in the nutritional supply and in the energy needs of an organism. Hence, strategies that maintain a steady state under varying nutritional conditions - referred to as energy homeostasis - are crucial for the healthy functioning of an individual. Inability to effectively maintain energy homeostasis results in the development of metabolic disorders like obesity, anorexia and diabetes. Complex metabolic syndromes, with the associated risk of cardiovascular diseases, afflict more than 34% of the adult population in the United States [1], making it expedient to dissect the molecular mechanisms underlying energy homeostasis.

Although *Drosophila *and humans diverged several hundred million years ago, significant insights have been derived from genetic studies in fruit flies. The genes that determine the body plan and development of fruit flies are evolutionarily conserved and have been found to be crucial also during the early development of human embryos [2,3]. Fundamental components of signaling pathways, including wingless, notch and hedgehog, were originally identified and characterized in fruit flies. Flies are long established as one of the prime models for research in developmental biology, cell biology and neurobiology, but it is only in the past decade that they have been significantly deployed to dissect energy metabolism. This lag is partly attributable to the lack of robust assays for lipid and carbohydrate metabolism and an under-appreciation of the extent to which *Drosophila *organ systems have functional analogues to vertebrate counterparts. This gap is being filled, however, by a number of recent studies that have led to the characterization of physiological roles for different organs in energy homeostasis in the fly (reviewed in [4-9]).

In this article, we summarize the rationale for using *Drosophila *as a model for the study of human metabolism, and then discuss in detail a few recent examples that have provided significant and novel insights into organismal metabolism.

## Parallels between *Drosophila *and human physiology

Many of the organ systems of flies are obviously analogous to those of their vertebrate counterparts: the gut absorbs nutrients, the fat body stores nutrients and functions as a nutrient sensor [10]. The *Drosophila *heart, a linear tube separated into four compartments by rudimentary valves, is essential for the circulation of nutrients and immune cells; but flies have an open circulatory system, rather than a vascular blood system, and oxygen is delivered by an independent tracheal system. This uncoupling allows specific aspects of metabolic dysfunction and heart function to be explored without compromising viability. Indeed, the fly has recently emerged as a model for the study of age-related heart dysfunction and polygenic cardiomyopathies (reviewed in [11,12]), providing insights into high-fat- and sugar-induced cardiac dysfunction [13,14].

The insect Malpighian tubules perform basic functions of the kidney such as transport, excretion, and osmoregulation, and fly nephrocytes, a second class of excretory cells present in the body cavity [15], are akin to vertebrate podocytes (cells in the kidney that ultra-filter blood to urine), and are crucial for dealing with metabolic stress and detoxification. It has been argued on the basis of gene expression profiling and the identification of nephrocytes that podocyte biology and renal function can be explored in the fruit fly system [16].

## Regulation of metabolism in *Drosophila *and mammalian systems

**Figure 1:**
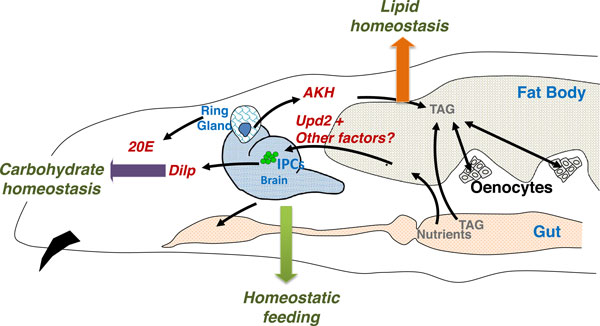
**Organs involved in metabolic homeostasis in *Drosophila***. Hormone names are in red, tissue names in blue, outputs in green. Figure adapted from Leopold and Perrimon [7].

Two key proteins, insulin and adipokinetic hormone (AKH, the fly glucagon) are responsible for regulating carbohydrate and lipid homeostasis. Insulin producing cells (IPCs) in the median neurosecretory (mNSC) region of the fly brain are akin to the pancreatic β cells. Corpora cardiaca (CC) cells located in the ring gland (neuroendocrine organ) secrete AKH and are reminiscent of pancreatic α cells (Figure 1). While insulin promotes the uptake of sugar by tissues from the blood (hemolymph in flies) and its storage as glycogen and fats, AKH is secreted during starvation conditions to break down glycogen and fats [17].

Flies express eight *Drosophila *insulin like peptides (Dilps), which are counterparts of the mammalian insulin and insulin-like growth factors. Among them, Dilp 2, 3 and 5 are expressed in the IPCs [18]. Under conditions of nutrient surplus, Dilps are released from the IPCs [19]. Consistent with their role in maintaining carbohydrate homeostasis, ablation of the IPCs results in elevated circulating sugars [20]. Similar developmental programs are involved in the specification of the IPCs in *Drosophila *and pancreatic β cells in mammals. IPCs develop from a pair of neural stem cells and their differentiation is under the control of the transcription factor Eyeless, whose mammalian homolog, Pax6, is also required for pancreatic beta-cell specification [21]. Deletion of *Dilp 1-5 *results in phenotypes that model type I diabetes, and include elevated circulating sugar levels, reduced stored fat and initiation of starvation responses, such as autophagy, despite adequate nutrition [22]. Finally, in adult flies, high fat diets (HFDs) and high sugar diets (HSDs) induce obesity and insulin resistance [23-25], suggesting that the molecular mechanisms of insulin resistance are conserved.

The fat body serves as a lipid store similar to the white adipose tissue in mammals. It stores lipids as triacylglycerol (TAG) packed into lipid droplets. A delicate balance between fat storage and breakdown is crucial to maintain energy homeostasis. Under conditions of nutrient deprivation, fat mobilization is triggered by the release from CC cells of AKH, which activates the G-protein-coupled receptor AKHR to promote lipolysis and glycogen breakdown [17,26]. Key components of lipolysis are evolutionarily conserved between mammals and *Drosophila*, as exemplified by studies on the lipase Brummer (Bmm), the ortholog of the primary mammalian lipase ATGL [27], which is required for basal levels of lipolysis. As with *ATGL *mutant mice, *Bmm *flies are obese and starvation-sensitive due to their inability to mobilize fat stores during starvation. Another key lipase in mammals is HSL (hormone sensitive lipase), which is required for lipolysis under starvation conditions; its *Drosophila *homolog plays a similar part in lipid mobilization [28]. The recruitment of lipases to lipid droplets is controlled by perilipin proteins that associate with lipid droplets to promote lipid storage and controlled lipolysis. Two perilipins have been characterized in flies [29]: Lsd-2 promotes fat storage as mutants of *Lsd-2 *are lean and starvation sensitive [30]; and *perilipin-1 *mutants display adult onset obesity and hyperphagia [31], suggesting a role for this molecule in controlled access of the lipid droplets to lipolytic enzymes. Together these studies suggest that *Drosophila *is a suitable system for the study of lipid metabolism.

Until recently, it was thought that fruit flies lacked the crucial human adiposity signal Leptin, believed to be a molecule exclusive to vertebrates. However, a JAK/STAT pathway ligand, Unpaired 2 (Upd2), has been shown to be the functional analogue of human Leptin, with a specific role in the fly fat body in the stimulation of insulin secretion in response to fats in the diet. Remarkably, the nature of the neural circuits through which Upd2 exerts its effects on insulin secretion is very similar to that of the modules on which Leptin acts [32], suggesting that questions pertaining to human Leptin biology can now be addressed in the fly.

In addition to the Leptin-like neuronal circuits, others that sense nutrients are present and have been proposed to be similar to the hypothalamic circuits in humans [33]. For example, a number of neural components that have parallels in mammalian brain, including neuropeptide F (NPF, ortholog of mammalian neuropeptide Y), serotonergic circuits and hugin neurons that affect feeding, have been discovered in flies [34-36]. Sophisticated behavioral assays have shown how signaling by NPF in olfactory sensory neurons couples hunger state to foraging behavior and how increased insulin signaling under conditions of nutrient surplus inhibits the motivation to feed [37]. Flies also have internal nutrient sensors that regulate food consumption on the basis of its nutritional value [38-40]. Fructose-sensing brain neurons have recently been shown to have a receptor called Gr43A that senses levels of fructose in the hemolymph, and controls feeding behavior in a satiation-dependent manner [41].

These examples highlight the parallels between the mammalian and fruit fly systems, and provide compelling evidence for the evolutionary conservation of metabolic regulation. In recent years, studies on fly metabolism have progressed from simply documenting the relevance of *Drosophila *to human biology, to providing novel insights on mechanisms underlying metabolic regulation. In the sections below, we give a few examples.

## New roles for translation inhibitor 4EBP in stress and lifespan extension

Several studies have established in various model systems (yeast to mice) that nutrient availability alters insulin signaling. The target of rapamycin (TOR) protein kinase is the growth regulatory target of insulin signaling in well fed animals [42,43]. TOR also cell-autonomously responds to amino acids, ATP and oxygen [44]. Thus, TOR signaling connects the intracellular state with the external nutritional status. One of the primary phosphorylation targets of TOR is the translation inhibitor 4EBP [45]. Previous work in mammalian tissue culture assays had implicated 4EBP as a growth regulator, but studies in flies have led to reconsideration of this model, because *4EBP *mutant flies show no abnormalities of development or organismal growth, but have severe defects of lipid metabolism [46]. Specifically, *4EBP *steady-state mRNA levels are up-regulated under conditions of stress or starvation, and *4EBP *mutants exhibit impaired survival under starvation. The starvation sensitivity of the *4EBP *mutants has been attributed to impaired fat mobilization, because the mutants burn their fat stores more rapidly than wild-type controls. These findings suggest that 4EBP acts as a metabolic regulator under stress conditions [47].

Evidence from studies on the extension of lifespan by dietary restriction in flies is also consistent with 4EBP as a metabolic regulator under conditions of stress [47]. Although it is ordinarily an inhibitor of translation, 4EBP can selectively increase translation in stress conditions. Indeed, the translation of genes encoding components of the mitochondrial electron transport chain, and thus mitochondrial activity, were found to be up-regulated by dietary restriction in a 4EBP-dependent fashion. The extension of lifespan by dietary restriction was abolished in *4EBP *mutant flies, in which the increase in mitochondrial activity failed to occur. Together, these results suggest that during nutrient stress, a 4EBP-mediated metabolic shift toward increased mitochondrial function prolongs lifetime [47].

## A neuronal role for S6K in nutritional choice and hunger driven behavior

The other primary target of TOR is the kinase S6K. In the presence of amino acids, S6K is activated by phosphorylation and promotes mRNA translation and growth. *Drosophila *studies have identified a novel role for S6K in neurons, revealing that it regulates nutrient choice [48,49] and hunger-driven behaviors [50]. All organisms, including fruit flies, choose between different nutritional sources (for example, carbohydrates versus proteins). In nutrient balancing assays - where flies were deprived of either sugars or proteins for three days and then were presented a diet that provided a choice between sugars or protein - flies selectively consumed nutrients that were lacking in their previous diet, thus regaining nutrient homeostasis. These studies also showed that activation of neuronal S6K was crucial for flies to make such a value-based nutritional decision.

## Altered lipid metabolism in human neurodegenerative disease models

Given the energy needs of neuronal cells, it is not surprising that deficits in energy metabolism manifest themselves most prominently in neuronal cell types. Genes that play a role in lipid homeostasis and mitochondrial function have been linked to adult onset neurodegeneration and have been extensively reviewed elsewhere [4,51,52]. Here we discuss insights obtained from a fly model of Friedreich's ataxia (FRDA) [53]. FRDA is the most common form of an autosomal recessive neurodegenerative disease affecting the central and peripheral nervous systems. It is caused by reduced expression of the mitochondrial protein frataxin, whose deficiency affects citric acid cycle function. Diabetes is a typical symptom of FRDA patients, and electron microscopic analysis of the neurons and cardiac muscles in mouse models shows an increase in lipid droplets [54], suggesting that there may be changes in lipid metabolism. To pursue further the role played by abnormal lipid metabolism in FRDA pathogenesis, *Drosophila *frataxin was removed from glial cells (neuronal support cells) by RNA interference (RNAi). This resulted in increased lipid droplet accumulation in glial cells and increased sensitivity to oxidative insults, neurodegeneration and impairment in locomotor activity. Interestingly, overexpression of *Glial Lazarillo *(*GLaz*) - the *Drosophila *homolog of human apolipoprotein D, a carrier protein of lipids - confers a protective effect on the *Frataxin-RNAi *flies. These studies suggest for the first time a specific requirement for frataxin in glial cells, and open the possibility that the control of lipid metabolism by apolipoproteins could represent a new strategy for the treatment of FRDA patients.

## The Warburg effect in *Drosophila *development

While the physiology of growth control in insects is very different from that of mammals, a recent study highlights an interesting parallel between metabolic regulation in *Drosophila *larval growth and that of tumor growth [55]. *Drosophila *goes through six different developmental stages - embryonic, three larval, pupal, and adult - stimulated by pulses of the steroid hormone ecdysone [56], which provide a recognized system for studying the interplay between metabolism, nutrition and development (see review [57]). The single *Drosophila *ortholog of the estrogen-related receptor, dERR, has been found to play a key role in carbohydrate and triacylglycerol metabolism. *dERR *mutants die as second-instar larvae with very high circulating sugar levels and very low triacylglycerol levels because of metabolic defects resulting from a decrease in aerobic glycolysis and pentose phosphate shunt. Hence, *dERR *promotes a proliferative metabolic program favoring the conversion of carbohydrates to biomass during larval development. This is reminiscent of the metabolic state of cancer cells, which use aerobic glycolysis to convert nutrients into biomass as opposed to energy, a metabolic adaptation to sustained growth known as the Warburg effect [58]. The authors of the paper point out that the three mammalian ERRs are associated with cancer progression, suggesting an ancestral framework for the association between ERRs and rapid growth.

## High-throughput screens to identify novel players in lipid metabolism

The power of the *Drosophila *system is the opportunity it offers to use high-throughput screens with robust assays to identify novel players in fundamental processes. This is well illustrated by recent genome-wide screens in both flies and in *Drosophila *S2 cells. For example, a genome-wide transgenic RNAi screen in adult flies for altered triglyceride levels identified a role for hedgehog signaling in adipocyte determination [2]. The screen identified a large number of genes already known to play a key part in mammalian fat or lipid metabolism, as well as a plethora of candidate genes for a role in the regulation of triacylglycerol storage, a large proportion of which had no previous annotated biological function. Another study [59] used buoyancy-based screen of *Drosophila *larvae to identify fat-storage mutants. Finally, a screen for regulators of lipid droplet formation and utilization in *Drosophila *S2 cells was the first to identify a requirement for functional Golgi vesicular transport in lipolysis [60]. The tools available in *Drosophila *to allow effective genetic screens are likely continue to identify novel regulatory mechanisms to inform investigations in mammalian systems.

In addition to the examples cited above, studies in *Drosophila *have identified links between the complex processes of immunity and circadian rhythms and that of organismal metabolism (reviewed in [5]). For example, the gut microbiota was recently found to influence host metabolism by impinging on TOR signaling [61]. Similarly, regulation of circadian factors by post-translational sugar modifications in response to nutrient status has been observed in fruit flies and found to be conserved in mice [62,63]. In the future, we can expect more contributions from such studies in *Drosophila *to inform us of how inter-organ communication signals contribute to organismal metabolic homeostasis.

## Note

This article is part of the *BMC Biology *tenth anniversary series. Other articles in this series can be found at http://www.biomedcentral.com/bmcbiol/series/tenthanniversary.
